# Biotic Stress-Induced Priming and De-Priming of Transcriptional Memory in *Arabidopsis* and Apple

**DOI:** 10.3390/epigenomes3010003

**Published:** 2019-01-14

**Authors:** Kay Gully, Jean-Marc Celton, Alexandre Degrave, Sandra Pelletier, Marie-Noelle Brisset, Etienne Bucher

**Affiliations:** IRHS (Institut de Recherche en Horticulture et Semences), UMR 1345, INRA, Agrocampus-Ouest, Université d’Angers, SFR 4207 QuaSaV, Beaucouzé F-49071, France

**Keywords:** benzo(1,2,3)thiadiazole-7-carbo-thioic acid *S*-methyl ester (BTH), epigenetics, plant protection, DNA methylation, transcriptomics

## Abstract

Under natural growth conditions, plants experience various and repetitive biotic and abiotic stresses. Salicylic acid (SA) is a key phytohormone involved in the response to biotic challenges. Application of synthetic SA analogues can efficiently prime defense responses, and leads to improved pathogen resistance. Because SA analogues can result in long-term priming and memory, we identified genes for which expression was affected by the SA analogue and explored the role of DNA methylation in this memorization process. We show that treatments with an SA analogue can lead to long-term transcriptional memory of particular genes in *Arabidopsis*. We found that subsequent challenging of such plants with a bacterial elicitor reverted this transcriptional memory, bringing their expression back to the original pre-treatment level. We also made very similar observations in apple (*Malus domestica*), suggesting that this expression pattern is highly conserved in plants. Finally, we found a potential role for DNA methylation in the observed transcriptional memory behavior. We show that plants defective in DNA methylation pathways displayed a different memory behavior. Our work improves our understanding of the role of transcriptional memory in priming, and has important implication concerning the application of SA analogues in agricultural settings.

## 1. Introduction

Plants are under continuous attack by pathogens because they are rich sources of nutrients. However, they protect themselves by physical barriers, such as a waxy cuticular layer or by protective periderm. In addition to these barriers, plants have evolved an immune system comprising constitutive and inducible defenses. The inducible immune system is based on the specific recognition of pathogen-derived molecules [1,2]. This so-called pattern-triggered immunity (PTI) is achieved by plasma membrane localized pattern-recognition receptors (PRRs), which directly interact with highly conserved pathogen/microbe-associated molecular patterns (PAMPs/MAMPs) [3,4]. One of the best-studied examples of a MAMP, and one that is capable of activating plant immunity, is the bacterial flagellin, which is the major component of the bacterial motility organ [5]. The perception system of flagellin is widely conserved across the plant kingdom, since most plants respond to flagellin [6]. The fastest MAMP-triggered defense responses are typically induced within 5 min and decrease within a 30 min time frame [7]. These events include the following plant responses: apoplastic alkalinisation, burst of reactive oxygen species (ROS), activation of mitogen-activated protein kinases (MAPKs), and intracellular calcium burst [8,9,10]. Late responses are induced within several hours to days and include the accumulation of callose, the inhibition of growth, the differential expression of defense genes, and the production of salicylic acid (SA) [9]. In line with short- and long-term defense responses, plants are also capable of inducing long-lasting systemic immunity. By local compatible or incompatible interactions, such systemic immunity can be initiated. This results in systemic acquired resistance (SAR). SAR-like responses can be induced by exogenous application of SA or SA analogues [11,12,13]. However, SA does not seem to serve per se as a mobile signal inducing immunity in uninfected distal tissues, while SA derivates or several other small molecules have been proposed to fulfil such a role [14].

Defense-related stimuli enhance the capacity of plants to activate defense responses [15,16]. Exogenous application of SA and other benzoic acid derivates have been shown to induce resistance of tobacco against the tobacco mosaic virus (TMV), and to cause the accumulation of pathogenesis-related (PR) transcripts [13]. This discovery paved the way for companies to identify more potent related compounds. These compounds are referred to as synthetic plant defense elicitors [17]. One of the most frequently used elicitors is benzo(1,2,3)thiadiazole-7-carbo-thioic acid *S*-methyl ester (BTH), which is commonly named acibenzolar *S*-methyl (ASM) and which is commercialized by Syngenta [18,19]. Application of BTH has been shown to restrain downy mildew infections in vegetables, and to control a range of fungal, bacterial, and viral diseases of important crops like tomato, cucumber, broccoli, tobacco, melon, and pear and apple trees [20,21,22,23]. In apple, it was shown that application can limit the extent of several diseases: apple scab, caused by the ascomycete fungi *Venturia inaequalis*, and the fire blight disease, which is caused by the bacterium *Erwinia amylovora* [24,25,26]. The SAR-inducing ability of compounds such as BTH is frequently associated with a primed state in which plants are able to ‘recall’ a previous infection or exposure to stress. Primed plants are therefore capable of responding more rapidly and/or effectively to a subsequent biotic or abiotic stress [16]. The molecular mechanisms behind priming are largely unclear. Priming has been associated with the accumulation of post-translational modification of cellular compounds. These compounds have important roles in signal transduction and/or amplification. In general, an accumulation or modification of these compounds does not activate a broad panel of plants’ defense responses [16]. Epigenetic regulation of gene expression is another widely discussed mechanism involved in defense priming. It has been shown that histone modifications at promotors of defense-related transcription factors such as *WRKY*6, *53*, and *29* contribute to priming of gene expression by BTH [27]. An additional epigenetic regulation mechanism is DNA methylation. In plants, DNA methylation is present in all three possible sequence contexts (CG, CHG, CHH, where H is A,T, or C) and has been shown to influence defense responses [28,29]. DNA methylation in the CG context can be maintained by DNA methyltransferase 1 (MET1), and in all sequence contexts, it can be triggered by the RNA-directed DNA methylation (RdDM) pathway. In RdDM, one of the main players is nuclear RNA polymerase D1 (NRPD1), the largest subunit of RNA Polymerase IV (Pol IV), which plays a key role in the initiation of siRNA production [30]. Another emerging regulation of gene expression involves antisense transcripts. It has been previously reported that genes transcribed in sense orientation can also be transcribed in antisense orientation. Antisense transcripts include partial or complete sequences complementary to other transcripts, and are endogenous RNA molecules [31]. They play an important role in various processes, including the adaptation to biotic and abiotic stresses [32]. Antisense transcripts are widespread in both prokaryotes [33] and eukaryotes [34].

Here, we show that stresses can lead to long lasting transcriptional memory (priming) and that priming can be reversed (de-priming) by subsequent stresses. Furthermore, our results show that antisense transcripts contribute to transcriptional memory, and that DNA methylation is required for proper stress response.

## 2. Results

### 2.1. BTH Induces Short- and Long-Term Defense Responses in Arabidopsis

**Figure 1:**
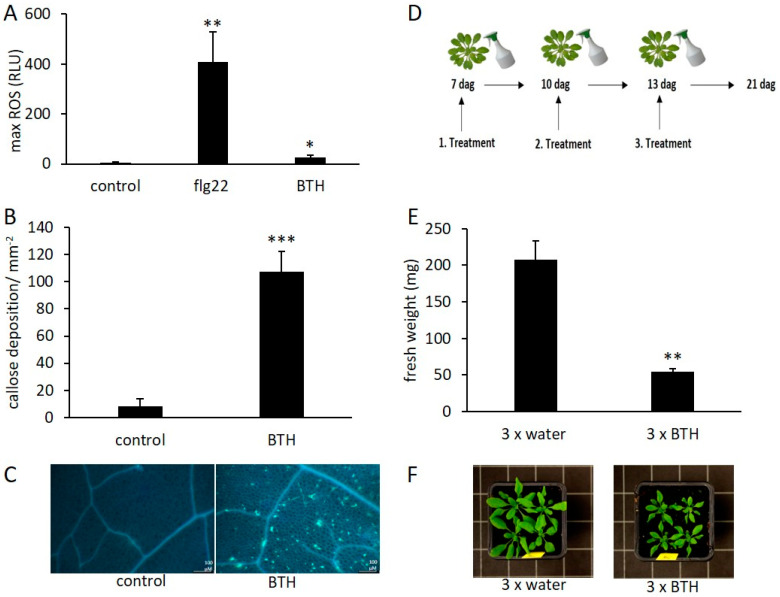
Benzo(1,2,3)thiadiazole-7-carbo-thioic acid *S*-methyl ester (BTH) induces short- and long-term defense responses in *Arabidopsis.* (**A**) Production of reactive oxygen species (ROS), measured in RLU (relative light units), in wild-type *Arabidopsis* leaf discs (Col-0), treated with 1 µM flg22, 1 mM BTH or without elicitor (control). Graphs display averages of 12 replications. (**B**) Quantification of callose deposition. The bars represent the mean of 4 replications. (**C**) Localization of callose deposition by aniline blue staining. (**D**) Temporal order of applied *Arabidopsis* treatments. The first treatment was applied 7 dag (days after germination), the third treatment 13 dag. (**E**) Quantification of fresh weight of 21 dag old *Arabidopsis* plants. Plants were previously sprayed three times with water or BTH (1 mM), according the scheme shown in (**D**). (**F**) Pictures of 21 dag plants previously sprayed with water or BTH (1 mM). Error bars show ± SE of the mean. Significant differences according to Student’s *t*-test results: *, *p* < 0.05; **, *p* < 0.01; ***, *p* < 0.001.

While it is known that bacterial elicitors, such as flg22, induce various defense responses in numerous different plants, less is known about the action of synthetic plant defense elicitors such as BTH. Here, we tested the short- and long-term responses of plants treated with BTH. One of the fastest plant defense responses is the accumulation of ROS [35]. Therefore, we first tested if a BTH treatment resulted in a detectable ROS burst in *Arabidopsis*. We found that BTH, applied at a final concentration of 1mM, induced a weak, albeit significant (*p*-value < 0.05) ROS burst, whereas a control treatment with flg22 lead to a strong accumulation of ROS (Figure 1A). One of the long-term defense responses is the deposition of callose. As shown by [36,37] BTH induces a deposition of callose in leaf tissue. We also observed that application of BTH lead to a strong accumulation of callose in *Arabidopsis* leaves (Figure 1B,C). Another long-lasting defense response is growth inhibition. Addition of bacterial elicitors to the growth medium was shown to result in a strong inhibition of growth [38]. Our results indicate that repetitive spraying with BTH led to similar effects. Seven days after germination (dag), young plants were sprayed with BTH and then treated two additional times at three day intervals (Figure 1D). Plants treated three times with BTH showed a reduction of fresh weight after a recovery phase of 8 days following the last BTH exposure, in comparison to the water control (Figure 1E,F).

**Figure A1:**
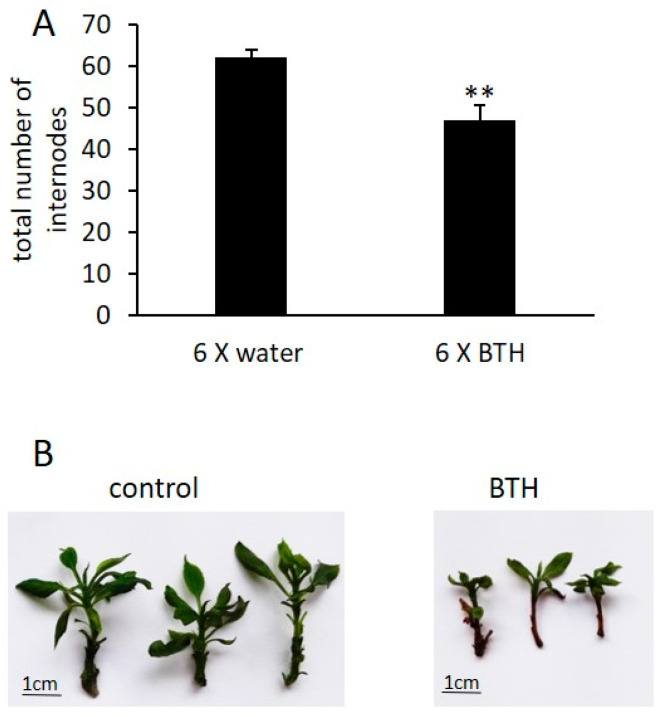
Growth repression effect of BTH on grafted and in vitro apple plants. (**A**) Total number of internodes of grafted apple plants treated six times with BTH (1 mM) or water. Two months after grafting, plants were treated every three days. Number of internodes was counted three days after the last treatment. Bars represent the mean of at least two biological replications. (**B**) Pictures of in vitro grown apple plantlets. After propagation plantlets were grown for 4 weeks after propagation on media supplemented with or without 1 mM BTH. Error bars show ± SE of the mean. Significant differences according to Student’s t-test results: **, *p* < 0.01.

To test the more general effect of BTH on other plants, we choose to investigate the growth repression effect, observed in *Arabidopsis*, on apple grown under two different growth conditions. Grafted apple plants exposed six times to BTH showed a lower number of internodes in comparison to a mock treatment (Figure A1A). Moreover, in vitro apple plantlets grown for 4 weeks on media supplied with BTH showed a strong inhibition of growth (Figure A1B).

### 2.2. Transcriptional Response and Memory Resulting from BTH Treatment in Arabidopsis

**Figure 2:**
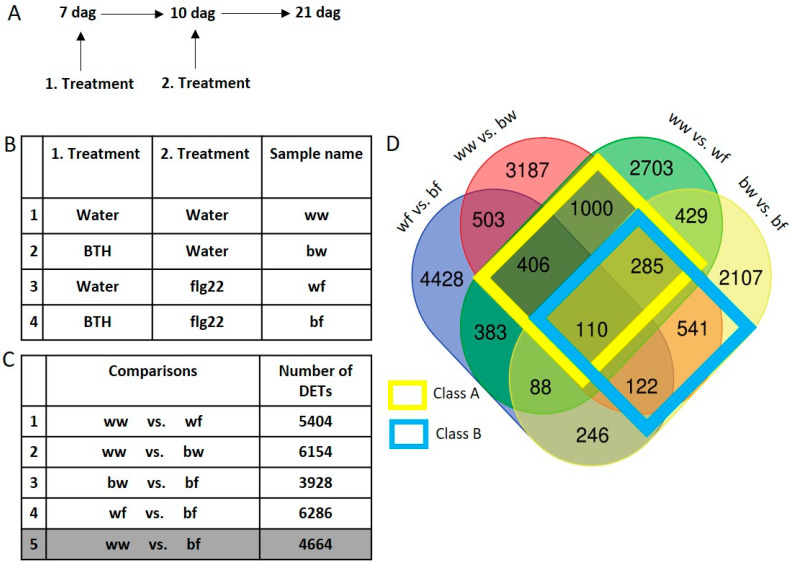
*Arabidopsis* transcriptome analysis set-up and results. (**A**) Experimental set-up of applied treatments. The first treatment was applied to young *Arabidopsis* plants 7 dag (days after germination), the second 10 dag and plants were harvested at 21 dag. (**B**) List of applied treatments after 7 and 10 dag as well as the sample name. (**C**) List of examined microarray comparisons and number of DETs. (**D**) Venn diagram showing DETs of all comparisons, and common DETs within the examined comparisons.

BTH is associated with inducing a primed state of gene expression [36]. In order to identify primed (and memorized) genes resulting from BTH application, we performed a series of microarray-based transcription profiles. Additionally, we investigated the role of a subsequent biotic stress exposure on BTH-induced long term transcriptional memory. To investigate this, we applied a series of treatments by spraying young *Arabidopsis* plants 7 dag. With a first application we primed gene expression with BTH. After a 3 day recovery phase, 10 day old plants were treated with the bacterial elicitor flg22. To test for long lasting effects, plants were harvested eleven days following the last treatment, and RNA extracted for subsequent gene expression profiling using two independent biological replications (Figure 2A). Four different treatment combinations were used: A control treatment where plants were sprayed two times with water (w) (sample name: ww), and three combinations whereby either BTH (b) or water (w) was used in the first treatment and water or flg22 (f) in the second treatment (sample names: bw, wf and bf, respectively, Figure 2B). Gene expression changes induced by these treatment combinations were assessed using microarrays. The long-term effects of flg22 or BTH were investigated by comparing these samples against ww (ww vs. wf and ww vs. bw, respectively). To determine the effect of flg22 after previous BTH application, we compared the samples bw against bf. The effect of BTH before a subsequent flg22 application was determined by comparing wf against bf. The impact of both compounds in succession was studied by comparing ww against bf (Figure 2C). In order to facilitate this complex analysis, differently expressed transcripts (DETs, which include antisense transcripts) were first represented in a Venn diagram. At this point we focused on the first 4 comparisons (Figure 2C). The visualization of DETs revealed that a substantial number of transcripts were only differently expressed in one comparison, and not in any of the others. Indeed, the number of DETs which were exclusively differently expressed in one comparison spanned from 2107 DETs (for bw vs. bf) to 4428 DETs (for wf vs. bf). 110 transcripts were found to be differently expressed in all four comparisons (Figure 2D). For our further investigations, we focused on the DETs which were differently expressed by BTH (ww vs. bw) and by flg22 (ww vs. wf). We categorized all common DETs of these two comparisons in Class A DETs (yellow box). Class A contained 1801 DETs. We were also interested in the effect of a subsequent exposure to flg22 after BTH treatment. These DETs can be found in Class B DETs (blue box). Class B contained common DETs detected in the comparison ww vs. bw and bw vs. bf, and comprises 1058 DETs (Figure 2D).

**Figure 3:**
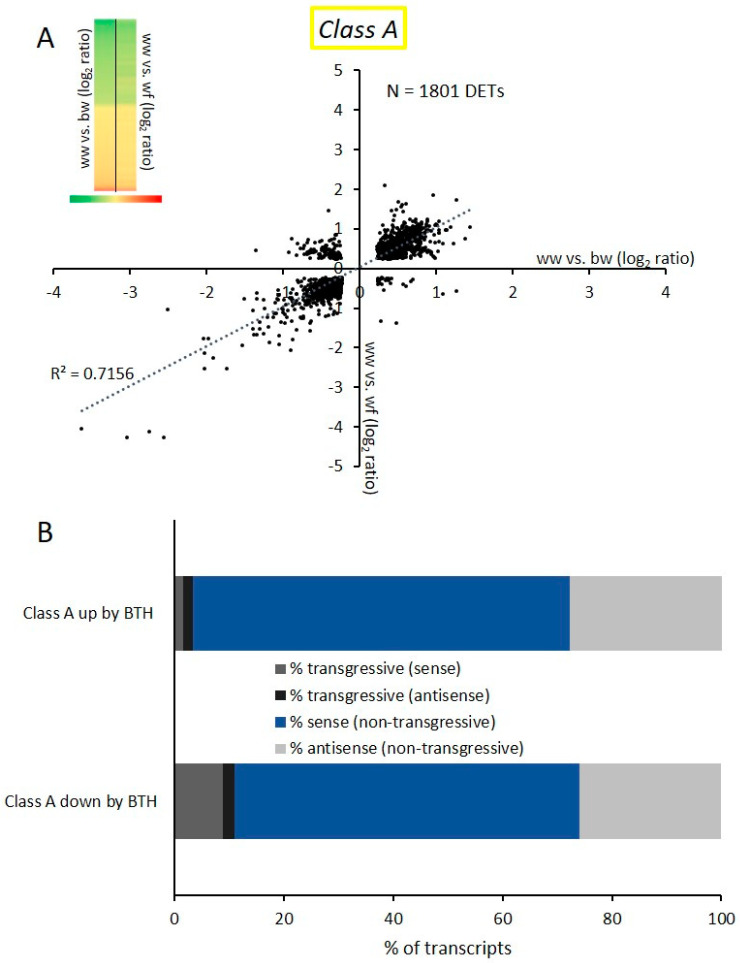
Common differentially expressed transcripts resulting from flg22 and BTH treatments. (**A**) Scatter plot of the log_2_ expression values of Class A DETs showing ww vs. bw on the X axes and ww vs. wf on the Y axes. The expression profile is also shown as a heatmap (upper left). DETs of ww vs. bw are shown in the first column and DETs from ww vs. wf in the second column. Up-regulated DETs are indicated in green and down-regulated DETs in red. (**B**) Class A was divided into Class A up by BTH and Class A down by BTH subcategories. The graph represents the percentage of non-transgressive transcripts expressed in sense and antisense in both sub-categories, as well as transcripts that showed a transgressive expression pattern (sense and antisense separated) and therefore did not follow the global expression trend.

Next, we analyzed Class A DETs in greater detail. This class compares the effects of BTH and flg22; we investigated transcripts that are commonly differently expressed following application of flg22 (ww vs. wf) and BTH (ww vs. bw). A scatter plot of the Class A DETs shows that the majority of common transcripts presented a similar expression profile (Figure 3A). Most transcripts that are up- or down-regulated by flg22 application were globally regulated in a similar fashion after BTH treatment (*R*^2^ = 0.7156). The 1801 DETs of Class A are also represented in a heatmap (Figure 3A). Next, we investigated DETs present in Class A in greater detail. Therefore, the category was divided into two sub-categories, up- and down-regulated by BTH. Indeed, only 3.4% of the 831 transcripts up-regulated by BTH (Class A up by BTH) were down-regulated by flg22, thus showing a transgressive expression profile. 1.6% of these transgressive transcripts were transcribed in sense orientation and 1.8% in antisense orientation. We observed a similar trend for the BTH down-regulated transcripts (Class A down by BTH) where only 10.9% were transgressive (8.9% in sense orientation and 2.0% in antisense orientation). We define DETs as ‘transgressive’ when these do not follow the global expression trend. In Class A, DETs with a transgressive expression profile are up-regulated by BTH but down-regulated by flg22, as well as DETs down-regulated by BTH but up-regulated by flg22. Both sub-categories showed a similar distribution of transcripts in sense and antisense orientation of non-transgressive transcripts, where 25% of all non-transgressive DETs were antisense transcripts (Figure 3B).

**Figure 4:**
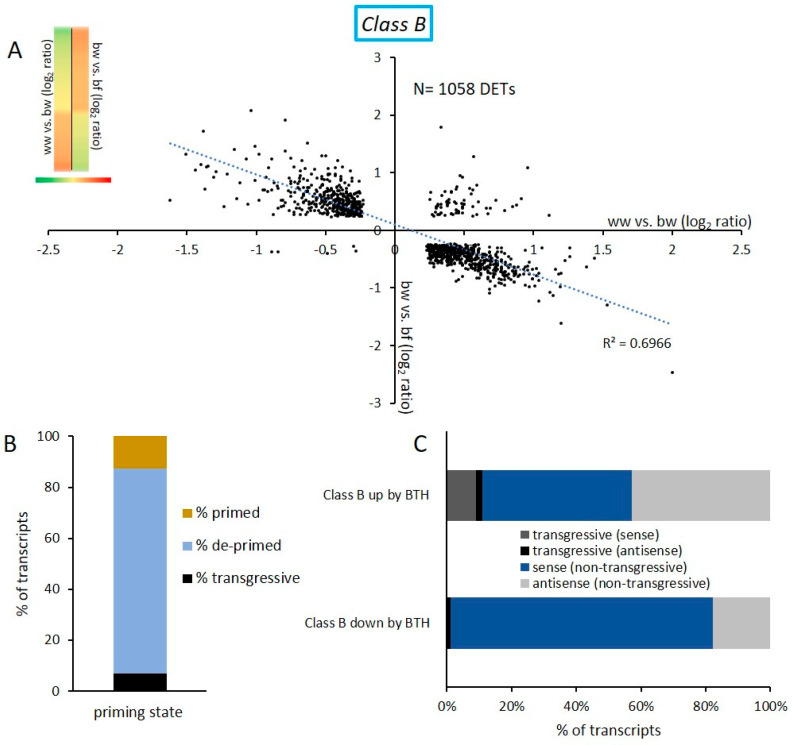
DETs of Class B show an inversion of gene expression profile following a second stress. (**A**) Scatter plot of transcripts present in the Class B DETs. The category contains DETs of the comparison ww vs. bw (*X* axes) and bw vs. bf (*Y* axes). Class B DETs are also shown with a heatmap (ww vs. bw iin the first column, and bw vs. bf in the second column) (**B**) Classification of transcripts present in Class B. The 1058 transcripts were compared to DETs of ww vs. bf. Results show 80.5% of the transcripts were not differently expressed by the combination of both treatments, and are considered as de-primed. 12.6% were differently expressed and therefore primed, and 6.9% did not follow the global expression trend in Class B. (**C**) Distribution of sense and antisense transcripts in the sub-categories Class B up by BTH and Class B down by BTH. The sub-category Class B up by BTH contains 42.9% non-transgressive antisense transcripts.

Next, we investigated the impact of subsequent stresses on BTH-induced transcriptional memory. Class B contains genes that were differently expressed after applying BTH alone and after applying flg22 after a previous BTH exposure (ww vs. bw and bw vs. bf, respectively). 1058 DETs were found in this class. The expression profile of these transcripts caught our attention because it showed that transcripts that were initially up-regulated by BTH were down-regulated by a subsequent flg22 exposure. Conversely, transcripts that were first down-regulated by BTH were up-regulated by subsequent flg22 treatment (Figure 4A). Only 6.9% of all transcripts in this category showed a transgressive expression profile (Figure 4B). A Fisher’s exact test revealed the high significance of the observed expression profile, with a statistical value of 0.00001. This suggested that subsequent stresses could erase the primed state of a substantial number of DETs. Therefore, we investigated how many of those DETs in Class B went back to a basal expression level. Because the application of BTH is closely related to the priming of genes ([36,39] and this study) we tested if a subsequent flg22 treatment led to a de-priming of genes. Here, we define de-priming as a memorized transcript expression returning to a basal (pretreatment-like) expression level by a subsequent stress. To assess the number of de-primed genes, we compared the transcription levels of DETs of Class B with those of the comparison ww vs. bf (Figure 2C). DETs of Class B that were not significantly differently expressed in ww vs. bf can be considered as de-primed. Out of the 1058 DETs of Class B, 80.5% were not significantly differently expressed in the comparison ww vs. bf anymore (p-value > 0.05) (Figure 4B). Only 12.6% of these transcripts maintained their priming state after the flg22 challenge.

Closer inspection of the non-transgressive Class B DETs revealed an uneven distribution of sense and antisense DETs. We investigated two sub-categories within this category: The sub-category Class B up by BTH contains DETs that were up-regulated by BTH and globally show the inverted expression by subsequent flg22 treatment. The sub-category Class B down by BTH contains DETs down regulated by BTH and up-regulated by subsequent flg22 treatment. While transcripts in the sub-category Class B up by BTH included 42.9% of antisense transcripts, transcripts in Class B down by BTH only contained 17.8% antisense transcripts and 81.0% sense transcripts (Figure 4C).

**Figure A2:**
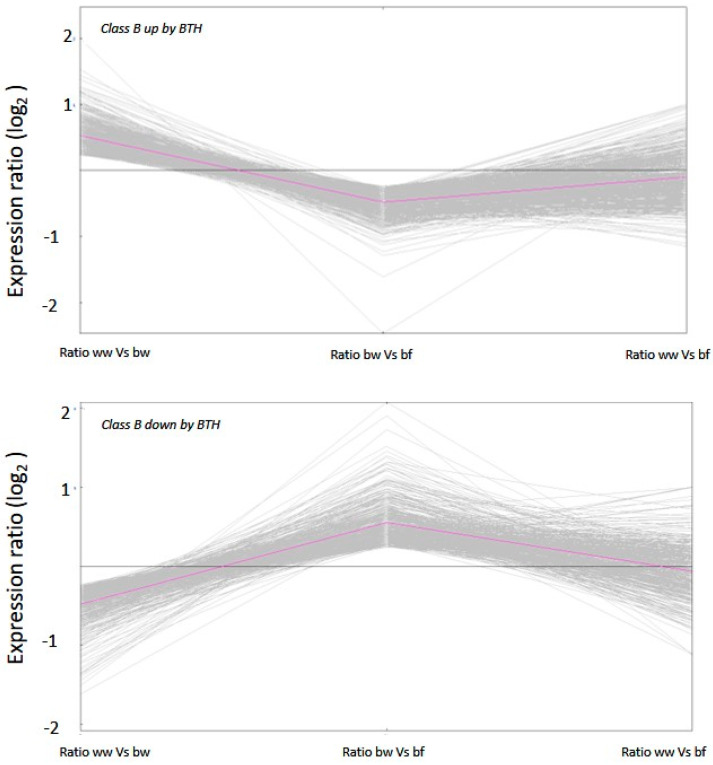
Different expression value plot of all Class B DETs, and the comparison ww vs. bf confirming the de-priming expression profile. Log_2_ expression values of DETs of the two sub-categories in Class B (transgressive DETs excluded), as well as their different expression value in the comparison ww vs. bf, were plotted.

In order to confirm the de-priming expression profile of the 80.5% DETs in the Class B, we plotted the different expression values of the two sub-categories Class B up by BTH and Class B down by BTH, as well their expression ratio in the comparison ww vs. bf. The expression profile confirms the de-priming expression pattern (Figure A2).

**Figure A3:**
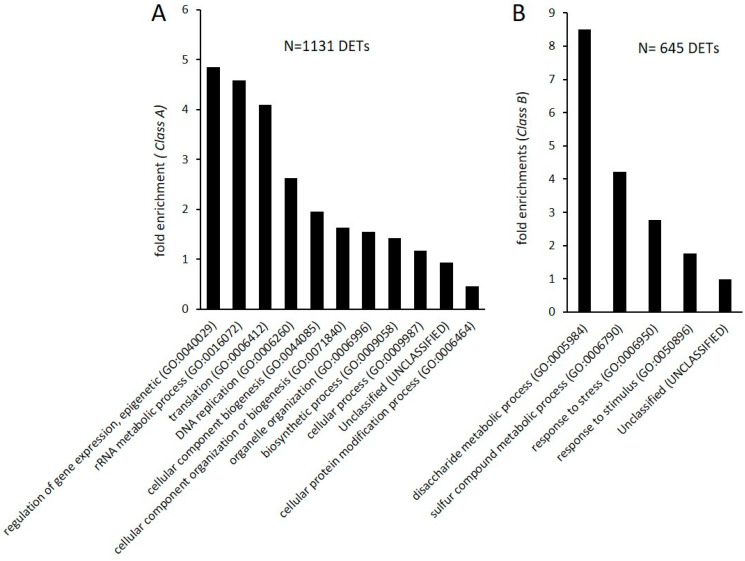
Gene ontology analysis of DETs in Class A and Class B. (**A**) PANTHER overrepresentation test of DETs in Class A. Gene ontology (GO) was available for 1131 out of the 1801 DETs. DETs correlated with the regulation of gene expression and epigenetics (GO:0040029) were overrepresented, with 4.85 fold enrichment compared to the *Arabidopsis* reference. (**B**) Overrepresentation test of Class B. Gene ontology for 645 of the 1058 DETs was available. GOs were enriched for disaccharide metabolic process, sulfur compound metabolic process, response to stress and stimulus (GO: 0005984, GO: 0006790, GO:0006950, GO:0050896) were overrepresented in this category. Gene ontology overrepresentation test was probed using the PANTHER webtool, using a Fisher’s exact test (Mi et al., 2017) [64].

Next, we studied the gene ontology overrepresentation of DETs in Class A and Class B (antisense transcripts included). DETs in Class A showed an overrepresentation in gene ontology correlated with the regulation of gene expression and epigenetics, rRNA metabolic process, and translation (Figure A3A), while DETs of the Class B were overrepresented in the gene ontology correlated with disaccharide metabolic process and sulfur compound metabolic process, as well as with response to stress and stimulus (Figure A3B).

### 2.3. Transcriptional Response and Memory of Stress Treatments in Apple 

**Figure 5:**
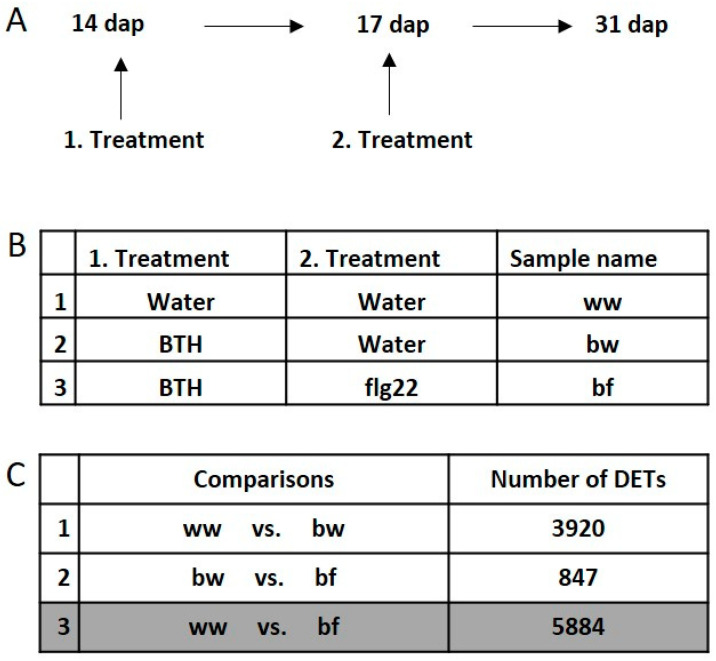
Whole genome expression profiling and applied comparisons in apple. (**A**) Experimental set up of apple plantlet treatments. Apple plantlets were treated the first time 14 dap (days after propagation) followed by one additional treatment 17 dap. Leave tissue was harvested 31 dap. (**B**) Combinations of applied treatments and sample names. (**C**) List of examined microarray comparisons and number of DETs.

Next, we wanted to test if our observations in *Arabidopsis* were also relevant for other plant species. For that purpose, we performed a similar treatment regime as for *Arabidopsis* on *in vitro* grown apple plantlets followed by microarray-based expression profiling using the latest version of the apple genome sequence [40]. Plants were treated 14 days after propagation (dap) and 17 dap. After 14 days of recovery plantlets were harvested, RNA extracted and subjected to transcription profiling (Figure 5A). In total 3 different treatment combinations, on two independent biological replicates were applied (Figure 5B). Similar to our experiments in *Arabidopsis* we explored the effect of BTH as a first treatment (ww vs. bw) as well the influence of flg22 application after BTH treatment (bw vs. bf). In order to identify DETs that returned to a basic expression level, we investigated the effect of a combination of BTH and flg22 treatment on apple plants (ww vs. bf) (Figure 5C). In the comparison ww vs. bw 3920 DETs were detected. Notably, these DETs result from a treatment 17 days before harvesting and RNA extraction. In the comparison bw vs. bf 847 DETs were found. 342 DETs are common in the comparison ww vs. bw and bw vs. bf. These common DETs are equivalent with the category Class B DETs of the *Arabidopsis* setup.

**Figure 6:**
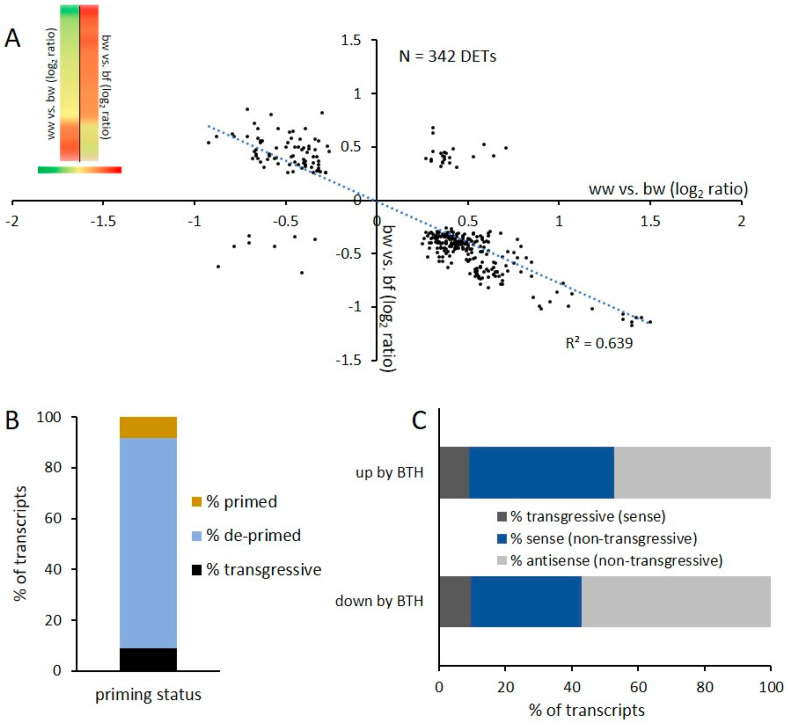
De-priming in apple. A: scatter Plots of common transcripts present in the comparisons ww vs. bw (*X* axes) and bw vs. bf (*Y* axes). A heatmap confirms the expression profile B: The common transcripts are compared to ww vs. bf. 82.7% are de-primed, 8.2% stay primed and 9.1% are transgressive and do not follow the global trend. C: The common DETs are divided into the sub-categories up by BTH and down by BTH.

The plot of the 342 common DETs confirmed our observations in *Arabidopsis* (Figure 6A). In total, 82.7% of these transcripts are de-primed and only 8,2% are still significantly differently expressed and therefore primed (Figure 6B). 9.1% of the transcripts are transcribed in a transgressive way (Figure 6B). Next, we determined how many transcripts lost their priming status by comparing the common DETs with the comparison ww vs. bf (Figure 5C). However, for apple an overrepresentation of antisense transcripts such as we have seen for *Arabidopsis* was not observed. The subcategory of common non-transgressive DETs which are up regulated by BTH showed 47.3% antisense transcripts and DETs down regulated by BTH are expressed with a 57.1% antisense contribution (Figure 6C). All transgressive transcripts in both subcategories are exclusively expressed in sense orientation. In total, common non-transgressive DETs have a higher content of antisense transcripts than found in our *Arabidopsis* comparisons. This observation is in line with a previous report that apple has globally a high percentage of antisense transcripts [41]. All *Arabidopsis* and apple comparisons are available in the Table S1.

### 2.4. DNA Methylation and De-Priming of Gene Expression

**Figure 7:**
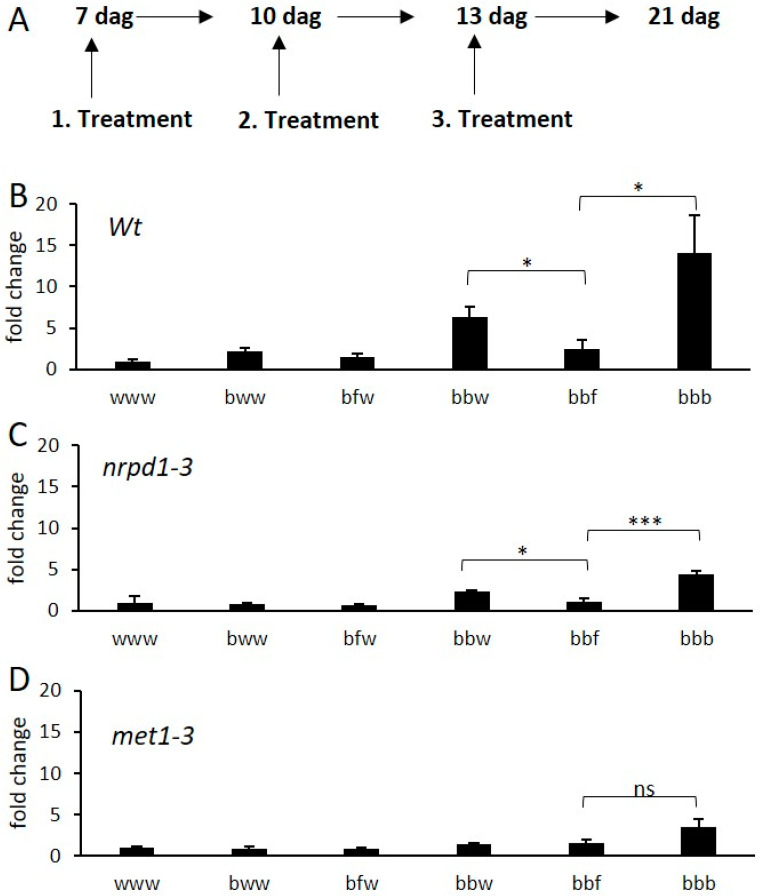
Expression of *AMY1* serves as marker for de-priming of transcription. (**A**) Set-up of treatments applied to *Arabidopsis* plants. The first treatment was applied 7 dag (days after germination), followed by two additional treatments. Plants were harvested 21 dag. (**B**) Expression profile of *AMY1* by different sequences of treatments of wild type plants. (**C**,**D**) The same treatment orders were applied to *nrpd1-3* and *met1-3*. All expression values were normalized to that of the gene *ACR12* (AT5G04740). Bars represent the mean of at least four biological replications. Error bars show ± SE of the mean. Significant differences according to Student’s t-test results: *, *p* < 0.05; ***, *p* < 0.001; ns: not significant.

**Figure A4:**
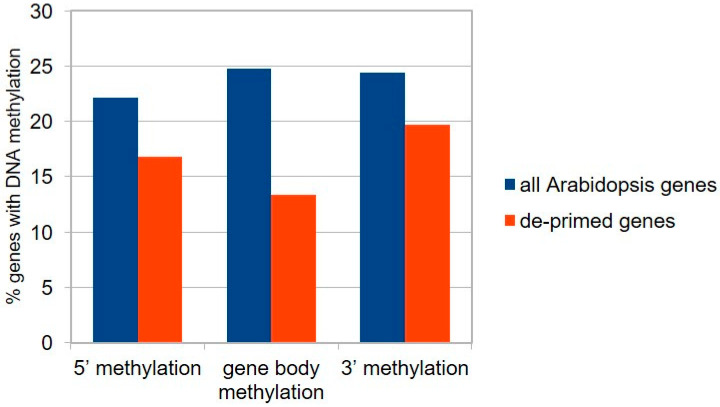
Class B DETs tended to be depleted in gene body methylation. Based on previously published data (Cokus et al., 2008) [44] genes were classed for the presence of DNA methylation in a promoter region (2 kb upstream, 5’ methylation), body of the genes (gene body methylation), and methylation downstream of the transcription end site (3’ methylation).

To investigate the mechanism behind the de-priming of genes by a second stress, we chose to follow the expression profile of *AMY1* (*At4G25000*), a gene found in the *Arabidopsis* sub-category Class B up by BTH. To enhance the contrast resulting from the treatments of *Arabidopsis*, we added a third treatment at 13 dag and harvested at 21 dag (Figure 7A). *AMY1* has previously been shown to be induced by biotic and abiotic stress, and to play an important role in starch metabolism [42,43]. Again, to be able to measure the long-term memory of *AMY1* transcription, sampling was performed eight days after the last treatment. In the sample bbf, *AMY1* was found to be significantly down-regulated in comparison to the bbw treatment, showing that we can follow de-priming by qPCR. Three BTH treatments (bbb) further enhanced the expression of *AMY1* in comparison to bbw and bbf (Figure 7B). However, using qPCR, we could not detect a significant differential expression of *AMY1* in the samples bww and bfw in comparison to the water control treatment. The expression profile of *AMY1* confirmed the previous observations with respect to the enhanced expression of this gene by BTH, and de-priming upon a subsequent flg22 exposure after BTH. Therefore, we used *AMY1* as a marker gene to investigate the molecular mechanisms involved in de-priming gene expression. To test if DNA methylation was involved in the memory or de-priming process, we applied the same combinations of treatments to plants defective in RNA-directed DNA methylation (*nrpd1-3*) and defective in the maintenance of DNA methylation during cell division (*met1-3*). For *nrpd1-3*, the expression profile was similar to that of wild type plants. However, the amplitude was four times lower for all samples (Figure 7C). The *met1* mutant did not show a significant response to any of the applied treatments, suggesting that *AMY1* activation and priming may directly or indirectly depend on DNA methylation. Looking for links between transcriptional memory and DNA methylation, we tested the correlation between Class B DETs and the DNA methylation status, using previously published methylomes [44]. Gene body DNA methylation was underrepresented in Class B DETs (13.4% genes methylated in the body compared to 24.7% at the whole genome level) (Figure A4).

## 3. Discussion

### 3.1. BTH Could Have Negative Effects on Plant Vitality in an Energy Trade-Off Balance

In their natural environment, plants are continuously exposed to a multitude of variable stresses. In an agricultural setting, plants such as apple need to be protected from various diseases with pesticides, since the lack thereof may cause tremendous declines in yield. In order to reduce the use of pesticides, compounds are now being developed that can enhance natural pathogen defense mechanisms in plants. Because BTH can contribute to defense priming ([27,45], and this work) it could have the potential to reduce yield loses. Here, we show that the application of BTH induced callose deposition and a ROS burst in *Arabidopsis* (Figure 1A,B). While other reports did not see this [37], this might be due to the tenfold higher BTH concentration of 1mM that was used in our assay. The induction of defense responses is widely connected to loss of energy in a trade-off balance [46]. In line with the results we show here, the application of BTH induced an inhibition of growth in both *Arabidopsis* and apple plants (Figure A1). These results indicate that BTH application at a higher concentration could have negative effects on plant growth.

### 3.2. De-Priming of Transcription Is Tightly Regulated

With the series of microarray experiments performed here, we found that flg22 and BTH treatments globally resulted in similar transcriptional changes in *Arabidopsis*. Class A (Figure 3) contained transcripts that were differently expressed by flg22 and BTH treatments; these transcripts showed the same trend in expression, as well as sense and antisense transcript distribution. BTH is an analogue of SA that is naturally produced by the plant [17]. SA is an important plant hormone that plays a role in the signaling pathway following flg22 perception [47]. Therefore, it is reasonable that common deregulated transcripts resulting from the two different treatments globally show a similar expression profile in *Arabidopsis*. We then tested how a subsequent stress affects BTH-induced transcriptional memory. We show that an flg22 treatment after BTH reversed the transcriptional memory in *Arabidopsis* (Figure 4A) and in apple (Figure 6A) of certain DETs. We termed such effects de-priming. It has previously been shown that BTH pre-treatments resulted in increased transcription of a subset of genes upon a second stress [27,48]. Indeed, a similar de-priming phenomenon has been observed for genes responding to repetitive drought stress. Liu et al., 2014 [49] showed that a subset of dehydration stress-response genes reacted to a first stress, but did not respond to a second stress and stayed at a basic non-stressed expression level. These genes are referred as ‘revised-response’ memory genes. Together with a follow-up work, it was shown that the transcription factor MYC2 plays a critical role for gene activation upon a second drought stress [49,50]. These results indicate that the regulation of de-priming in plants could depend on precise regulation of such a single gene. With our work, we show that the number of de-primed transcripts by a subsequent exposure to a second biotic stress reflected the majority of commonly regulated transcripts (Figure 4B and Figure 6B). It is remarkable that we only found 6.9% of DETs in the *Arabidopsis* Class B and 9.6% of DETs for apple that showed a transgressive expression profile and did not follow the global trend of de-priming (Figure 4B and Figure 6B). Therefore, the number of transgressive DETs is low. The total number of DETs of the comparison bw vs. bf in *Arabidopsis* and apple respectively is also notable: this comparison represented the lowest number of DETs in comparison to all other examined microarrays of both plant species in this study (Figure 2C and Figure 5C). This indicates that the global level of transcription was reduced by the subsequent flg22 treatment after BTH exposure.

### 3.3. DNA Methylation Could Contribute to the Priming Properties of BTH

Two plant memory mechanisms have been proposed: the accumulation of proteins or transcription factors is one of them, epigenetic changes is a second potential mechanism [51]. Our results show a strong effect on priming and de-priming by global methylation decrease. We identified *AMY1* in the *Arabidopsis* Class B as a good marker for transcriptional memory because: (1) it has been shown that AMY1 is a secreted protein that is expressed following biotic and abiotic stresses [42], (2) it shows a memory accumulating property following multiple treatments (Figure 7B), and (3) it has a de-priming property upon a succession of BTH and flg22 treatments (Figure 7B). Notably, we found that *AMY1* activation was strongly reduced in *met1-3*, only being activated after three subsequent BTH applications. This suggests that DNA methylation may be required directly or indirectly to activate and/or prime *AMY1*. However, due to the weak activation of *AMY1* in *met1-3*, the de-priming effect of flg22 after BTH treatment could not be observed with this method (Figure 7D). We thus cannot conclude whether *MET1* is required for the activation or the maintenance of the primed state of *AMY1*. In *nrpd1-3* we found a low expression value, but still detected the de-priming expression profile, indicating that DNA methylation via the RdDM pathway may be required to achieve the full potential of BTH treatments. However, we noted that globally, genes showing a de-priming expression pattern were rather depleted of DNA methylation. Overall, we concluded that DNA methylation may be contributing to the priming and de-priming events, possibly in an indirect fashion.

### 3.4. De-Priming Could Limit the Impact of Sequential Stresses

**Figure 8:**
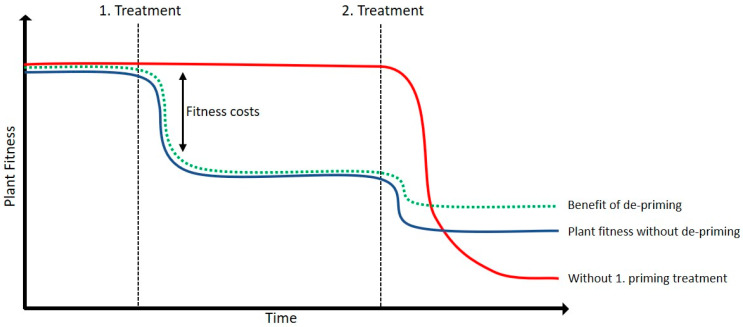
Proposed model of de-priming effect on plant fitness. Here we suggest a model of a possible beneficial effect of transcriptional de-priming. If plants are not exposed to a first priming treatment, the second treatment might cause stronger deficits on plant fitness (**red line**) in comparison to plants that have been primed (**blue line**). However, priming reflects a fitness costs for plants due to the induced defense response and the maintenance of the primed transcription and/or epigenetic memory. The second treatment could cause a less pronounced fitness cost in comparison to the un-primed plants. We propose that the de-priming of a DET subset could lead to an additional positive effect on plant fitness, by fine tuning the plant defense response and returning non-beneficial DETs to a basic expression level (**dashed green line**).

It has been shown that treatment with BTH increases the expression of the flg22 receptor FLS2 as well as the closely related co-receptor BAK1 [37]. An increased expression of BAK1 was shown to have strongly reduced growth and extensive cell death [52]. It might be possible that the subsequent flg22 exposure after BTH could fine tune the plant expression profile to reduce priming-related fitness costs. In line with this hypothesis is the gene ontology analysis of the Class B in our *Arabidopsis* comparisons, which revealed that DETs are overrepresented in the response to disaccharide and sulfur compound metabolic processes, as well as in response to stress and stimulus (Figure A3B). Interestingly, it was shown that several carbohydrates play an important role in plant immunity and plant protection [53]. This indicates that especially genes correlated with defense responses and stress responses return to a basic expression level. However, because of insufficient annotations of the apple genes, we could not compare these results with our apple comparisons. In Figure 8, we propose a model of the effect of expressional de-priming. We hypothesize that the de-priming of a subset of transcripts could enhance plant fitness. The second treatment and the resulting de-priming of transcripts could prevent negative effects of the priming treatment and/or fine tune the plant response to the more recent stress. It remains to be investigated if de-priming has an effect on plant resistance induction and/or fitness. It was shown with several examples that plants deal with stressful situations by inducing silencing mechanisms via antisense transcription, and that endogenous siRNAs derived from a pair of sense and antisense transcripts can enhance the tolerance to various stresses [54,55,56,57,58]. The strong overrepresentation of antisense transcripts in the *Arabidopsis* sub-category Class B up by BTH is in line with our hypothesis, in which plants induce de-priming in order to prevent negative or unnecessary effects of the priming stimulus. These antisense transcripts were up-regulated by BTH and down-regulated by the subsequent flg22 treatments. Therefore, it may be that enhanced antisense expression suppresses negative effects that the BTH treatment may have on the plant. With our work we describe a rather unusual expression profile caused by subsequent stresses in *Arabidopsis* and apple, in which plants have an efficient way to memorize stresses by transcriptional priming, and that such priming can readily be erased (de-primed) by subsequent stresses. The fact that our observations were made in both apple and *Arabidopsis* suggests that priming and de-priming may be conserved in the plant kingdom. These results have implications for the application of priming compounds in the field, as plants are constantly subjected to stresses, which may affect the efficiency of such compounds.

## 4. Material and Methods

### 4.1. Plant Material

Plant material used was wild-type *Arabidopsis thaliana* L. Heynh cultivar 6 Columbia (Col-0), as well as *met1-3* [59] and *nrpd1-3* [30]). All *Arabidopsis* plants were grown under long day condition (photoperiod of 16 h, light at 22 °C/ 8 h light at 22 °C, with 60% relative humidity). Apple cv. Golden Delicious double haploid (GDDH13, described by [60]) in vitro grown plantlets were propagated on MS (Murashige and Skoog) based medium with BA (6-Benzylaminopurine) 0.25 mg/L and IBA (Indole-3-butyric acid) 0.1 mg/L under short day conditions (photoperiod of 8 h light at 22 °C/16 h dark at 21 °C). Grafted apple plants (GDDH13) were grown in the greenhouse on a MM106 rootstock. Dormant buds were grafted in winter (February). After 10 days in a cold chamber they were potted in 1 L pots, and grown in normal greenhouse conditions until they developed to about 30 nodes.

### 4.2. Quantification of the Growth Inhibiting Effect of BTH

The growth inhibiting effect of BTH (Bion 50WG, Syngenta, Basel, Switzerland) was examined in grafted apple plants. Two months after grafting the plants were treated six times at three day intervals with BTH (1 mM) by applying the solution with a paintbrush on all leaves and the meristem. The total number of internodes was counted 3 weeks after the first treatment. The growth inhibiting effect of BTH on apple plantlets was observed by growing plantlets after propagation on MS (Murashige and Skoog) based medium with 0.25 mg/L BA, 0.1 mg/L IBA and 1 µM BTH, or the control medium without BTH for four weeks before the pictures were taken.

### 4.3. Transcriptomic Analysis

Four *Arabidopsis* plants represent one biological replication. Plants were treated with flg22 (QRLSTGSRINSAKDDAAGLQIA, obtained from Eurogentec SA (Angers, France)) or BTH by spraying with final concentrations of 1 µM and 1mM, respectively. Leaves of 20 apple plantlets represent one biological replication. Plantlets were treated by dipping the whole plantlet into filter sterilized flg22 (1 µM) and BTH (1 mM) solution, and placed on fresh growth medium after every treatment. Microarray analysis was performed for *Arabidopsis* with the CATMA IRHS array ((V1) GPL25797), and for apple with the *Malus domestica* array ((V1) (GPL25795)). Leaves of *Arabidopsis* and apple were collected from two independent biological replications. The RNA was extracted using the NucleoSpin RNA plant extraction kit (Machery-Nagel, Hoerdt, France) according the manufacturer’s recommendations. For *Arabidopsis* samples the Message AmpII aRNA amplification kit (Ambion) (Thermo fisher scientific, San Jose, CA, USA) and for apple samples the Low Input Quick Amp Labeling Kit, two-color (Agilent, Foster City, CA, USA) was used for cDNA synthesis and hybridization. The hybridizations were performed on a NimbleGen Hybridization System 4 (mix mode B) at 42 °C overnight. Afterwards, the slides were washed, dried, and scanned at 2 µm resolution. NimbleGen MS 200 v1.2 software was used for microarray scans, and the Agilent Feature Extraction 11.5 software was used to extract pair-data files from the scanned images. Statistical analysis was based on a dye switch approach as described in [61]. All statistical analyses were performed using the R language (R Development Core Team, 2009); data were normalized with the lowess method, and differential expression analyses were performed using the lmFit function and the Bayes moderated t test using the package LIMMA [62] from the Bioconductor project. Differently expressed transcripts, sorted by applying the binary code, were selected for a *p*-value < 0.05. Transcriptome data are available at Gene Expression Omnibus, with the accession GSE123073 for the *Arabidopsis* comparisons and GSE123072 for the apple comparisons.

### 4.4. Determination of Gene Expression by qPCR

4 plants per biological replication were harvested, frozen, and ground in liquid nitrogen. RNA from 100 mg of tissue was extracted using the NucleoSpin RNA plant extraction kit (Macherey-Nagel, Hoerdt, France). The DNase treatment was performed according to the manufacturer’s recommendations. Per PCR reaction, complementary DNA was synthesized from 10 ng of total RNA extract with oligo(dT) primers, using Moloney Murine Leukemia Virus Reverse Transcriptase according to the manufacturer’s instructions (Promega, Madison, WI, USA). For quantitative real-time reverse transcription PCR (qPCR) in a 96-well format, the Chromo4™ System (Bio Rad, Marnes-la-Coquette, France) was used. Expression was normalized to *ACR12* (AT5G04740) using the qGene protocol [63]. The primers used are as followed: *ACR12* (AT5G04740) acr12FW: TTGTTCGATGATCGCCGGAA, acr12REV: TGGAACAACGTCGTCATCGT; *AMY1* (At4G25000) amy1FW: AATACGGTTCAGAGGCGGAA, amy1REV: CGGAAGTCCCACCTTCGAAA.

### 4.5. Measurement of Reactive Oxygen Species

For ROS assays, leaf discs of three weeks old soil grown plants were placed into each well of a white 96-well plate (Thermo Scientific, Waltham, USA) in 0.1 mL of water and kept in the dark overnight. For elicitation and ROS detection, horseradish peroxidase and luminol were added to a final concentration of 10 µg/mL and 100 µM, respectively. Luminescence was measured directly after addition of concentrated BTH solution (final concentration of 1 mM) in a FLUOstar OPTIMA plate reader (BMG LABTECH, Offenburg, Germany).

### 4.6. Callose Deposition

Leaf discs were vacuum infiltrated for 10 min with BTH (1 mM) solution or water, and kept floated in the solution for 24 h. Afterwards, leaf discs were fixed and de-stained in 1:3 acetic acid/ethanol until leaf tissue was completely transparent. After washing the leaf discs in 150 mM K_2_HPO_4_ for 30 min, the plant material was stained for 2 h in 150 mM K_2_HPO_4_ and 0.01% aniline blue. Callose deposits were quantified with a Leica DM1000 microscope equipped with a Qimaging Micropublisher 3.3 RTV camera using a DAPI filter.

## Supplementary Materials

The following are available online at http://www.mdpi.com/2075-4655/3/1/3/s1. Table S1: List of all DETs and classes of Arabidopsis and apple comparisons.
